# Association of sex and APOE ε4 with brain tau deposition and atrophy in older adults with Alzheimer's disease

**DOI:** 10.7150/thno.48522

**Published:** 2020-08-21

**Authors:** Shaozhen Yan, Chaojie Zheng, Manish D Paranjpe, Jian Li, Tammie L.S. Benzinger, Jie Lu, Yun Zhou

**Affiliations:** 1 Department of Radiology, Xuanwu Hospital, Capital Medical University, Beijing, China.; 2 Mallinckrodt Institute of Radiology, Washington University in St. Louis School of Medicine, St. Louis, MO, USA.; 3 Harvard-MIT Program in Health Sciences and Technology, Harvard Medical School, Boston, MA, USA.; 4 Department of Neurology, Washington in St. Louis University School of Medicine, St. Louis, MO, USA.

**Keywords:** Alzheimer's disease, Tau PET, Sex, APOE, Neurodegeneration

## Abstract

The objective of this study was to assess the association of sex and the apolipoprotein E (APOE) ε4 allele with brain tau deposition and atrophy in older adults with Alzheimer's disease (AD) using quantitative ^18^F-AV-1451 positron emission tomography (PET) and magnetic resonance imaging (MRI).

**Methods:** Preprocessed ^18^F-AV-1451 tau PET, raw T1-weighted structural MR images, demographic information, cerebrospinal fluid (CSF) total tau (t-tau) and phosphorylated tau (p-tau) measurements from 57 elderly individuals with AD were downloaded from the Alzheimer's Disease Neuroimaging Initiative (ADNI) database. An iteratively reblurred Van Cittert partial volume correction (PVC) method was applied to all preprocessed PET images. MRI images were used for PET spatial normalization and gray matter volume calculation. ^18^F-AV-1451 PET standardized uptake value ratio (SUVR) was calculated relative to the cerebellum gray matter. The effect of sex and APOE ε4 status on SUVR and gray matter volume were assessed at both region of interest (ROI) and voxelwise levels.

**Results:** Female APOE ε4 carriers (FACs) had significant higher ^18^F-AV-1451 SUVRs in the lateral temporal, parietal, posterior cingulate, medial temporal, inferior temporal, entorhinal cortex, amygdala and parahippocampal gyrus regions, and exhibited smaller gray matter volumes in the posterior cingulate, medial temporal, inferior temporal and amygdala regions, as compared to the non-FACs (NFACs) comprised of female APOE ε4 non-carriers, male APOE ε4 carriers and male APOE ε4 non-carriers. Voxelwise analysis revealed forebrain and limbic clusters with greater ^18^F-AV-1451 SUVRs and lower gray matter volume between FACs compared to the NFACs. Negative correlations between ROI ^18^F-AV-1451 SUVRs and gray matter volumes were significant after adjusting for age and years of education.

**Conclusions:** Among elderly individuals with AD, sex modified the effects of the APOE ε4 allele on region-specific tau deposition and gray matter volume. FACs had elevated brain region-specific tau PET SUVR and decreased gray matter volume in comparison to NFACs. The study provides a basis for the use of precision medicine in the diagnosis of AD and evaluation of therapeutics using ^18^F-AV-1451 PET and structural MRI.

## Introduction

The apolipoprotein E (APOE) ε4 gene has emerged as a major genetic risk factor for Alzheimer's disease (AD) 1-3. APOE ε4 is associated with AD-related biological markers including cerebrospinal fluid (CSF) total tau (t-tau), phosphorylated tau (p-tau), brain tau deposition, and gray matter atrophy 4-9.

In addition to APOE ε4, sex also plays an important role in AD risk, with females having a higher lifetime risk of developing AD than males 10, 11. Sex differences in brain tau pathology and neurodegeneration have been identified in healthy older adults and patients with AD 12-14. The Lancet Neurology Commission has recently asserted that greater attention to sex differences in AD is essential to advancing both disease prevention and treatment strategies in AD 15.

Numerous CSF and epidemiological evidence support modulatory effects of APOE ε4 on sex-specific disease risk and neuropathology in AD. For example, female APOE ε4 carriers (FACs) have a greater risk of developing AD 16, 17 and far more pronounced disease progression 18. Studies of CSF t-tau and p-tau have suggested that FACs accumulate more tau pathology than males in clinically normal older adults 19, patients with mild cognitive impairment (MCI) 18, and even mixed diagnostic cohorts (clinically normal older adults, MCI and AD) 13. In addition, emerging evidence suggests that the cortical tau measured by quantitative positron emission tomography (PET) is temporally and prospectively related to the degree of neurodegeneration and current cognitive status 20-23. Two recent studies have investigated how sex modulates the effect of APOE ε4 on brain tau deposition using quantitative^ 18^F-AV-1451 PET. One study focused on clinically normal individuals in a 2-cohort study, and showed a significant sex by APOE ε4 interaction effect on regional tau retention 12. Our previous work reported a significant APOE ε4 by sex interaction effect on regional brain tau deposition in individuals with MCI 24. These two studies suggest that FACs exhibit brain regions with increased tau deposition among cognitively normal older adults and individuals with MCI.

Cross-sectional structural magnetic resonance imaging (MRI) studies have shown a similar APOE ε4 interaction effect on gray matter atrophy. In MCI, the effect of APOE ε4 on hippocampal volume reduction was stronger in females versus males 25. Similarly among AD patients, FACs had significantly smaller hippocampal volume compared to female APOE ε4 non-carriers, which, however, was not observed in males 9.

Understanding these APOE ε4 by sex interaction effects may help us identify and apply customized prevention strategies for different populations against AD. The evidence of how sex in association with APOE ε4 affects tau PET and gray matter volume, and whether tau pathology is associated with neurodegeneration in patients with AD remain poorly understood. The objective of the present study was to assess whether sex modulates the effects of the APOE ε4 allele on brain tau deposition and atrophy in older adults with AD.

## Methods

### Participants

We included 57 participants from the Alzheimer's Disease Neuroimaging Initiative (ADNI) who had (i) a clinical diagnosis of AD, (ii) undergone ^18^F-AV-1451 tau PET, (iii) high resolution 3D T1-weighted structural MRI, and (iv) APOE ε4 genotyping information. Consent was obtained from all participants prior to the start of the study. For each subject, the most recent tau PET and T1-weighted MRI scan were included. A full list of study inclusion and exclusion criteria can be found at https://adni.loni.usc.edu/wp-content/uploads/2008/07/adni2-procedures-manual.pdf.

### PET and MRI data acquisition and processing

Preprocessed ^18^F-AV-1451 PET images and raw T1-weighted images were downloaded from the ADNI database (http://adni.loni.usc.edu/). The ^18^F-AV-1451 PET acquisition parameters can be found at http://adni.loni.usc.edu/methods/pet-analysis-method/pet-analysis/. The PET images had been previously aligned, averaged, reoriented and interpolated into a standard image space with uniform voxel size (image volume 160 × 160 × 96, 1.5 × 1.5 × 1.5 mm^3^ in x, y, z), and then were smoothed to a uniform resolution of 8 mm in full width at half maximum (FWHM) by the ADNI consortium.

As described in our previous studies 24, 26, we further processed the downloaded PET images for partial volume correction (PVC) and spatial normalization using Statistical Parametric Mapping (SPM12, Wellcome Department of Imaging Neuroscience, Institute of Neurology, London, UK) with CAT12 toolbox (CAT12: http://www.neuro.uni-jena.de/cat/) and MATLAB R2019b (The MathWorks Inc). PVC was applied to the processed PET images to correct for or minimize potential underestimation in PET quantification. In brief, an iterative reblurred Van Cittert method 27 was used for PVC on the PET images. The algorithm was implemented using a 3-D Gaussian kernel with 8 mm FWHM, step length α =1.5, and an iteration stop criterion of relative percent change of PVC images < 1%. All PET images with PVC were then coregistered to the corresponding structural MRI images. The MRI images were normalized to standard Montreal Neurologic Institute (MNI) space using SPM12 with a MRI template (image volume: 121×145×121, voxel size: 1.5×1.5×1.5 mm^3^ in x, y, z). The transformation parameters determined by MRI spatial normalization were then applied to the coregistered PET images for PET spatial normalization. Regions of interest (ROIs) including cerebellum gray matter as a reference tissue were manually drawn on the MRI template using PMOD (version 4.002; PMOD Technologies Ltd., Zürich, Switzerland) in standard MNI space. SUVR images were calculated relative to the cerebellum gray matter. SUVR images without PVC were also generated for reference.

Braak Stage related 12 ROIs, including the entorhinal cortex, parahippocampal, amygdala, inferior temporal, medial temporal, lateral temporal, posterior cingulate, posterior precuneus, parietal, occipital, orbital frontal, and prefrontal, were also defined in the MNI space 22, 24, 26. The SUVR of each ROI was obtained by calculating the mean of the values within each ROI on the PVC and without PVC PET images in the MNI space. The choice to compute ROI SUVRs in standard space was made to minimize variance related to the variability of ROI volume and shape in native space 24, 26, 28, 29.

Gray matter volume was calculated using the CAT12 toolbox extension of SPM12. CAT12 preprocessing was conducted following the standard default procedure suggested in the manual. First, structural T1-weighted MRI images were segmented into gray matter, white matter and CSF. Then, gray matter segmentations were warped to MNI space with modulated spatial normalization. Finally, the 12 cortical ROIs gray matter volumes were calculated from the modulated gray matter images.

### APOE genotyping, CSF t-tau and p-tau assessment

A 10 ml sample of peripheral blood was collected from each subject for direct APOE genotyping. Restriction enzyme isoform genotyping was performed on the extracted DNA to test for the presence of the APOE ε4 genotype, as described previously 30. APOE ε4 carriers were defined as participants who had one or more ε4 allele (ε4/ ε4, ε4/ ε3 and ε4/ ε2). Those without any ε4 allele were defined as APOE ε4 non-carriers. CSF samples were acquired through lumbar puncture as described in ADNI: http://adni.loni.usc.edu/methods/documents/. The Roche study protocol developed by the UPenn/ADNI Biomarker Laboratory was used for Aβ42, t-tau, and p-tau CSF quantification, as described in previously 31, 32.

### Statistical analysis

Recent studies have found no significant differences among non-FACs (NFACs, including female APOE ε4 non-carriers, male APOE ε4 carriers and male APOE ε4 non-carriers) in ^18^F-AV-1451 ROI SUVRs in cognitively normal adults 12 and individuals with MCI 24. In the current tau PET study, we first tested the hypothesis that previous results from cognitively normal and MCI cohorts hold true in our AD cohort. Statistical analysis using generalized linear models (GLMs) proc with LS-means/pdiff, implemented in Statistical Analysis System (SAS version 9.4, SAS Institute, Inc.), confirmed that ^18^F-AV-1451 SUVR did not significantly differ among NFACs in any of the 12 study ROIs. Specifically, no regions were significantly different in an overall test of significance between NFAC groups with *P* values ranging from 0.19 for the medial temporal to 0.99 for the occipital. Likewise, in pairwise intergroup comparisons (i.e. female APOE ε4 non-carriers vs. male APOE ε4 carriers, female APOE ε4 non-carriers vs. male APOE ε4 non-carriers, and male APOE ε4 carriers vs. male APOE ε4 non-carriers), no regions were significantly different with *P* values ranging from 0.09 for the inferior temporal female APOE ε4 non-carriers vs. male APOE ε4 non-carriers in comparison to 0.99 for the posterior precuneus female APOE ε4 non-carriers vs. male APOE ε4 carriers comparison. Based on this finding, in order to increase statistical power, all subsequent analyses compared FACs to the NFACs. SAS9.4 and SPM12 were used for all statistical analyses. GLMs adjusted for age and years of education were used to assess group differences in SUVR and gray matter volume for both ROI and voxelwise analyses. For ROI analyses, *P* < 0.05 was considered significant. For voxelwise analyses, an uncorrected *P* < 0.001 and cluster size > 100 voxels were defined as significant. Additional statistical results with multiple comparison corrections are discussed in the Discussion section. To investigate whether regional tau retention was associated with neurodegeneration, the correlation between ROIs ^18^F-AV-1451 SUVRs and gray matter volume was analyzed using partial correlation analysis.

## Results

### Demographics

A total of 57 AD subjects (mean age: 78.98 ± 9.22 years, 25 females (44%), 34 APOE ε4 carriers (60%)) with ^18^F-AV-1451 PET and structural MRI scans were included in the study. Demographic characteristics, cognitive function and CSF Aβ42, t-tau and p-tau levels of FACs and NFACs are listed in Table 1. There were no significant differences in age and years of education between males and females for either the APOE ε4 carrier group or the non-carrier group (*P* > 0.05). Performance on the Mini-Mental State Examination (MMSE), global Clinical Dementia Rating (CDR) and CDR-sum of boxes (CDR-SOB) score (*P* > 0.05) were not significantly different among the four subgroups, after adjusting for age and years of education. Chi-squared analysis revealed no significant difference in the proportion of individuals with APOE ε4 ε4/ ε4 ε3/ ε4 ε2 genotypes (*P* = 0.95) between males and females.

### Sex modifies the APOE ε4 effect on brain tau deposition measured by ^18^F-AV-1451 PET SUVR

**Figure 1:**
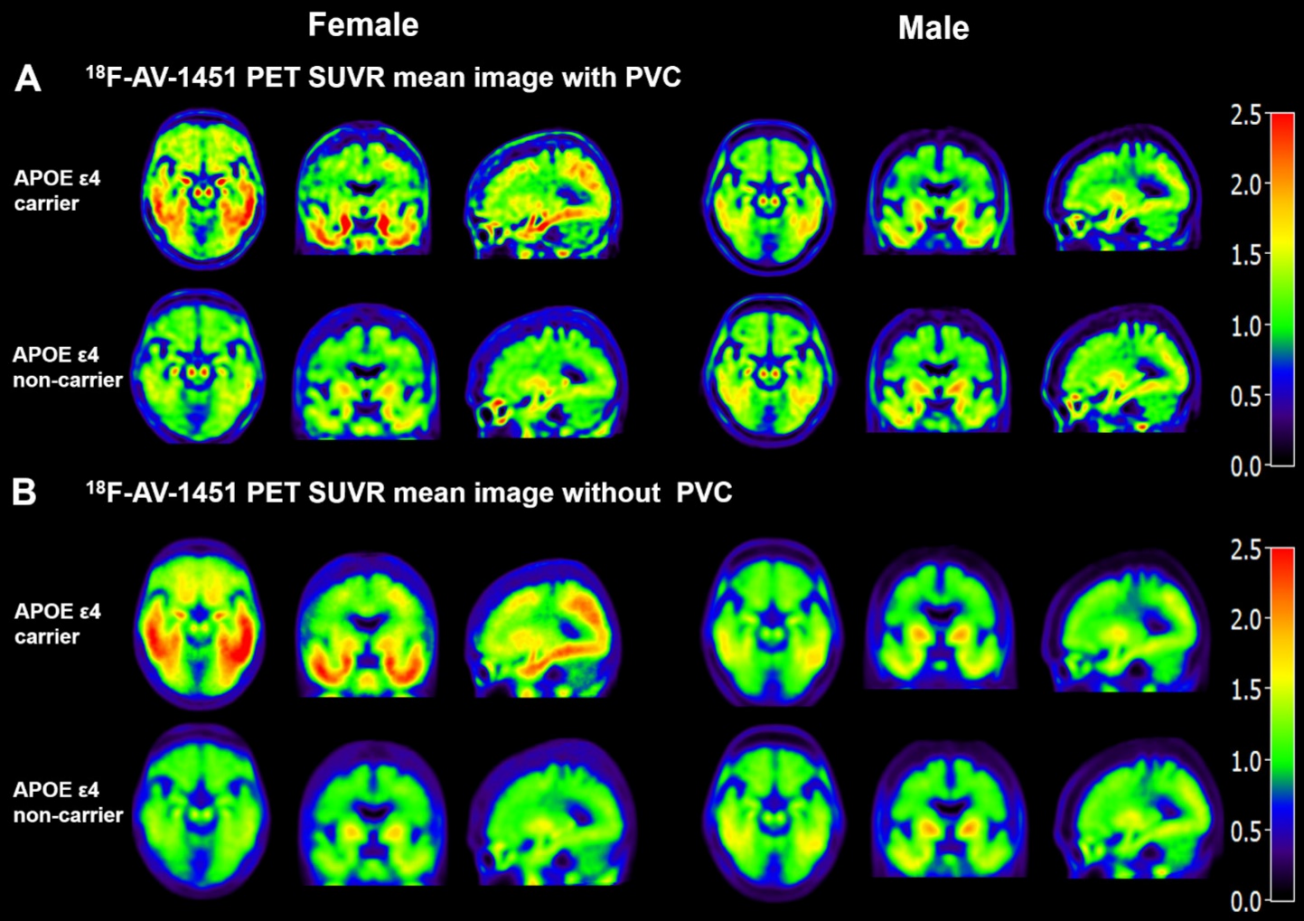
** Mean SUVR images of^ 18^F-AV-1451 PET with and without partial volume correction in individuals with AD.** Partial volume corrected SUVR images (**A**) show increased contrast and spatial resolution compared with the images without PVC (**B**). Both with and without PVC images visually demonstrate that FACs have higher ^18^F-AV-1451 SUVR. FACs: female APOE ε4 carriers, PVC: partial volume correction.

Mean ^18^F-AV-1451 SUVR images in Figure 1 visually demonstrate that FACs have higher SUVR in both PVC and without PVC images in comparison the NFACs. Compared to the without PVC images (Figure 1B), the mean ^18^F-AV-1451 SUVR images with PVC in Figure 1A exhibit a higher SUVR contrast between APOE ε4 carriers and non-carriers, consistent with our previous study 22. Based on this finding, PVC images were retained for subsequent analyses.

**Figure 2:**
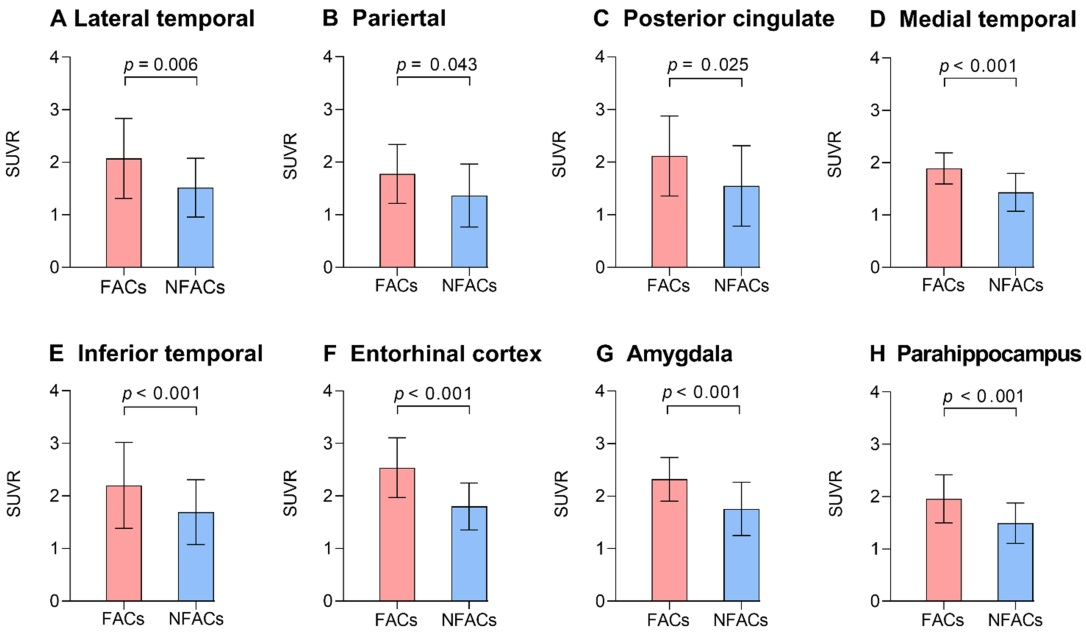
** The FACs have higher ROI ^18^F-AV-1451 PET SUVRs relative to NFACs with AD.** Mean (±standard deviation) of ROI ^18^F-AV-1451 PET SUVR for FACs (red) and NFACs with AD (blue) are depicted. *P* value was determined by a generalized linear model with age, years of education included as covariates. FACs: female APOE ε4 carriers, NFACs: non-FACs, SUVR: standardized uptake value ratio.

**Figure 3:**
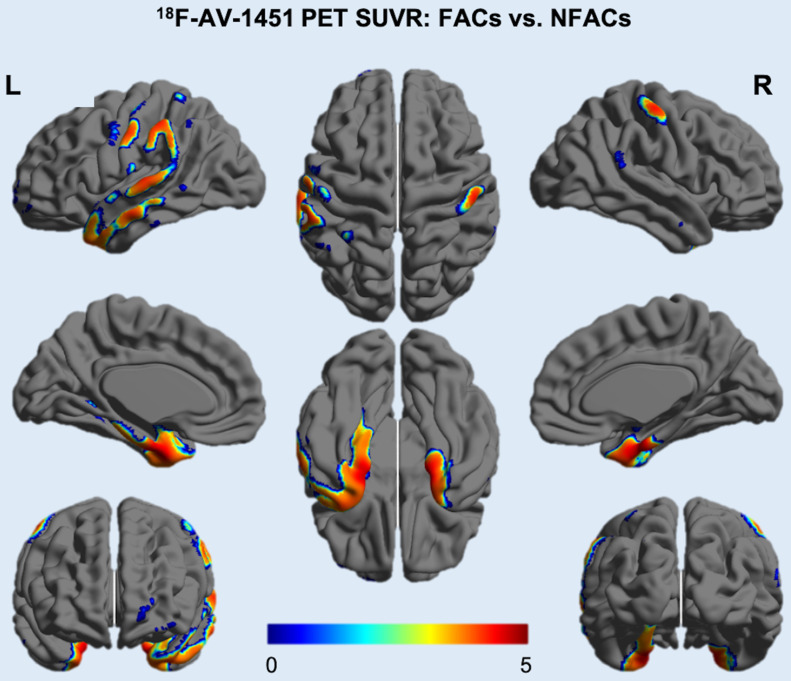
**^18^F-AV-1451 SUVR voxelwise difference between FACs and NFACs with AD.** T values are expressed in blue-red colors from 0 to 5 (*P* < 0.001, uncorrected, adjusted for age and years of education). FACs: female APOE ε4 carriers, NFACs: non-FACs.

After adjusting for age and years of education, ROI analysis revealed significantly higher ROI SUVRs between FACs compared to the NFACs with AD in the lateral temporal, parietal, posterior cingulate, medial temporal, inferior temporal, entorhinal cortex, amygdala and parahippocampal gyrus (*P* < 0.05; Figure 2). Voxelwise analysis revealed higher SUVR between FACs compared to the NFACs with AD in clusters corresponding to the bilateral middle temporal, middle temporal pole, inferior temporal, entorhinal cortex, parahippocampal gyrus, amygdala, left superior temporal, parietal, middle occipital, precentral, middle frontal, superior frontal and middle orbitofrontal lobe (*P* < 0.001, Figure 3, Table 2) after adjusting for age and years of education.

### Sex modifies the APOE ε4 effect on gray matter volume measured by structural MRI

**Figure 4:**
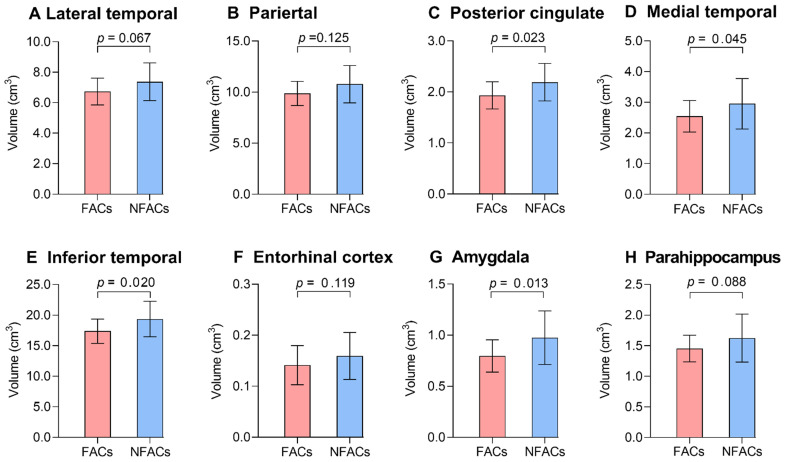
** The FACs had lower ROI gray matter volume relative to NFACs with AD.** Mean (± standard deviation) of ROI gray matter volume for FACs (red) and NFACs (blue) with AD are depicted. *P* values were determined by a generalized linear model with age, years of education were included as covariates. For consistency, the regions displayed are those in which FACs have significantly higher ROI ^18^F-AV-1451 PET SUVRs from Figure 2. FACs: female APOE ε4 carriers, NFACs: non-FACs, SUVR: standardized uptake value ratio.

**Figure 5:**
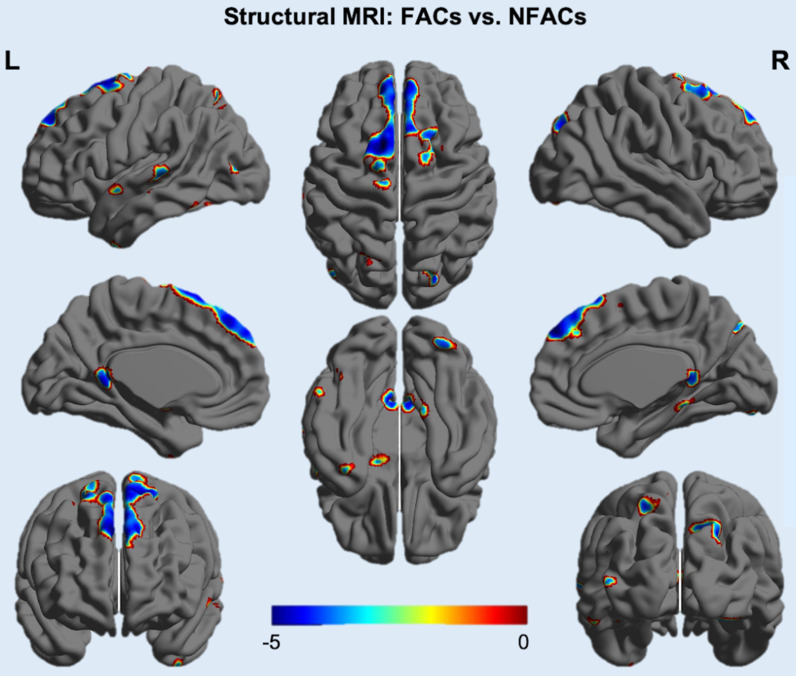
** Voxelwise difference of gray matter density between FACs and NFACs with AD.** T values are expressed along the blue-red scale from -5 to 0 (*P* < 0.001, adjusted for age and years of education). FACs: female APOE ε4 carriers, NFACs: non-FACs.

ROI-based analysis revealed significantly lower gray matter volume among FACs compared to the NFACs in the posterior cingulate, medial temporal, inferior temporal and amygdala after adjusting for age and years of education (*P* < 0.05; Figure 4). In voxelwise analysis, FACs had decreased gray matter volume in clusters located in the left inferior temporal, inferior occipital, middle temporal, middle occipital, amygdala, posterior cingulate; right superior temporal pole, superior occipital, precuneus, bilateral superior-medial frontal, supplementary motor area and superior frontal lobe compared to the NFACs after adjusting for age and years of education (*P* < 0.001, Figure 5, Table 3).

### Association between tau SUVR and gray matter volume

**Figure 6:**
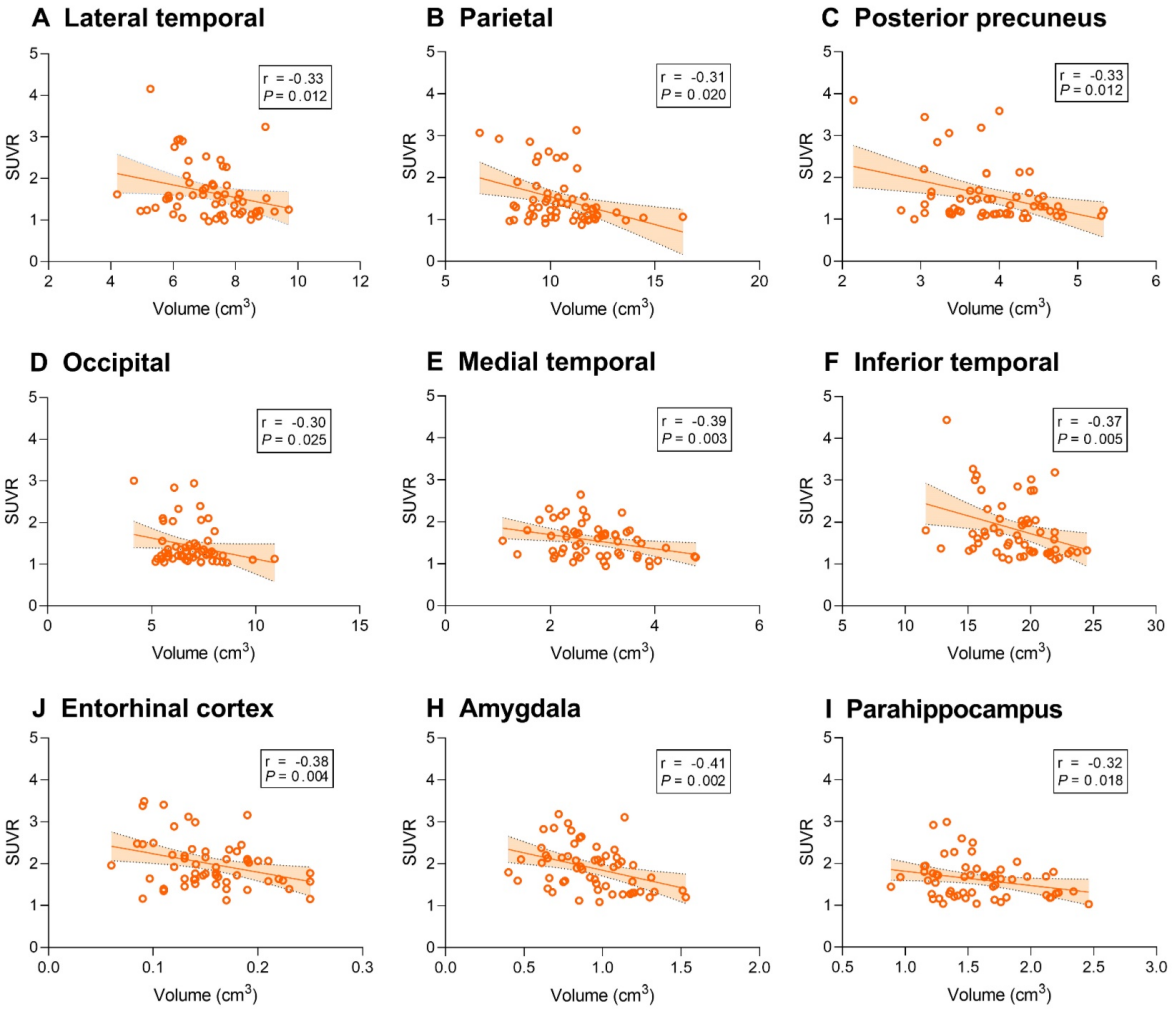
** Correlation between regional ^18^F-AV-1451 SUVR and gray matter volume in AD participants.** Line graphs showing correlation between regional gray matter volume (cm^3^) and ^18^F-AV-1451 SUVR. Fitted lines,* P*-values, and 95% confidence intervals are displayed from partial correlation analysis. SUVR: standardized uptake value ratio.

We then sought to identify associations between tau deposition and gray matter volume. ^18^F-AV-1451 SUVR was associated with gray matter volume of the temporal, parietal, posterior precuneus, occipital, entorhinal cortex, amygdala and parahippocampal regions (*P* < 0.05, Figure 6).

## Discussion

By extending previous work on APOE ε4-sex interaction effects on brain tau deposition in cognitively normal and MCI cohorts 12, 24, our study revealed that sex also modifies the effect of APOE ε4 on brain tau deposition and atrophy in individuals with AD using data from the ADNI consortium. Recent work on clinically normal individuals from the Harvard Aging Brain Study found no sex-APOE ε4 interaction effect on regional tau deposition. However, within the ADNI cohort, a sex by APOE ε4 interaction effect on brain tau deposition was previously found in the early Braak stages (Ⅰ-Ⅲ) regions including the entorhinal cortex, inferior temporal, amygdala, fusiform and parahippocampal gyrus 12, 33. For individuals with MCI, sex-stratified analysis showed that FACs had higher tau SUVR in the entorhinal cortex, amygdala, fusiform, parahippocampal gyrus, posterior cingulate, occipital, temporal, parietal and posterior precuneus (Braak stages Ⅰ-Ⅴ) compared to NFACs 24. In this study, we focused on older adults with AD, and found that FACs had more tau deposition in the temporal, parietal, post cingulate, entorhinal cortex, amygdala, parahippocampal gyrus (Braak stages Ⅰ-Ⅴ) regions compared to the NFACs. Overall, our results add to a growing body of work demonstrating an APOE ε4 and sex interaction effect in AD whereby FACs exhibit greater brain region-specific tau deposition compared to NFACs across sub-clinical and clinical stages of AD.

Recent metabolic and transcriptomic data add potential mechanistic explanations to the findings of this study 34-37. For example, prior work has found that specific metabolic effects were limited to FACs. A recent study found that, among FACs, higher acylcarnitine C10 was associated with higher CSF p-tau, and higher proline levels were associated with reduced brain glucose uptake. These effects were not observed in the NFACs, suggesting that FACs experience greater impairment of mitochondrial energy production compared to NFACs with AD 36. This provides an additional line of support for the findings drawn in this work. Moreover, in-vitro and transcriptomic data has suggested that sex modulates neuroinflammation, another risk factor for AD, with inflammatory dysregulation being stronger in females with AD 37, 38. APOE ε4 also triggers neuroinflammatory cascades that cause neurovascular dysfunction resulting in leakage of blood derived toxic proteins into the brain 39. These data suggest a potential model of AD pathogenesis in which the female sex and APOE ε4 allele contribute to neuroinflammatory damage to the brain and brain microvasculature, leading to increased tau and brain atrophy in FACs.

Strikingly, although FACs had greater tau deposition, we observed no differences in MMSE score, global CDR and CDR-SOB between FACs and NFACs. The implication that FACs exhibit increased tau deposition without commensurate cognitive decline warrants further investigation. This may suggest that FACs experience greater cognitive resilience to the effects of tauopathy compared to males 24. This is supported by a ^18^F-FDG PET study in which cognitively normal female adults had a younger metabolic brain age compared with males 40.

We also evaluated sex by APOE ε4 interaction effects on brain region-specific atrophy. FACs had a significant lower gray matter volume in the cingulate, medial temporal, inferior temporal and amygdala cortex than the NFACs with AD after adjusting for age and years of education. Previous studies have demonstrated that APOE ε4 allele is associated with significantly lower hippocampal gray matter volume in females with MCI and AD, but not in males 9, 25. Consistent with past findings, our ROI-based and voxelwise analysis showed that FACs had reduced gray matter volume in several cortical regions compared with the NFACs. Moreover, we also found negative correlations between brain regional tau PET uptake and grey matter volume, which is consistent with existing studies 41, 42. This suggests that tau pathology is a major driver of local and distant cortical atrophy 20, 43.

In the current study, we pooled data from female APOE ε4 non-carriers, male APOE ε4 carriers and male APOE ε4 non-carriers into a group labeled NFACs. To evaluate whether similar results persist when using not pooled data, we also analyzed group comparisons for ^18^F-AV-1451 SUVRs between FACs and female APOE ε4 non-carriers, male APOE ε4 carriers, male APOE ε4 non-carriers respectively. The results from both “not pooled” and “pooled” data were highly consistent across 6 out of the 8 originally significant ROIs including the lateral temporal, medial temporal, inferior temporal, entorhinal cortex, amygdala and parahippocampal gyrus. In the “non-pooled” data, the SUVRs of posterior cingulate and parietal showed a marginal *P* value between FACs and female APOE ε4 non-carriers, male APOE ε4 carriers, male APOE ε4 non-carriers (posterior cingulate: *P* = 0.07, 0.10, 0.08 and parietal: *P* = 0.12, 0.13, 0.09, respectively), but significant group differences were observed after “pooling” the data (*P* = 0.025 and 0.043). The findings suggest that the increased sample size from pooling data enhanced the study's statistical power to discover FAC-associated effects.

Limitations of this study must be considered when interpreting the results. First, our analysis was an observation study within the multicenter ADNI cohort. The high biological and physiological heterogeneity in AD related to age, sex and disease stage, and the relatively small number of AD subjects (15 FACs and 42 NFACs, all of whom received ^18^F-AV-1451 tau PET scans and APOE ε4 genotyping) may limit the generalizability of results from the study. For example, in our cohort the age range was 55-93 (78.98 ± 9.22 years) and CDR range was 0.5-2.0. Given that the effects of sex and APOE ε4 are known to vary significantly across disease stage and age groups 44, 45, our results should be interpreted within the age range in this cohort. It should be noted, however, that the frequency of FACs was 26.3%, which is comparable to an existing study 46. Further, due to its robustness within smaller sample sizes, a GLM was used to assess group differences in tau deposition and brain atrophy at both ROI and voxelwise levels. We also evaluated the probability that the current results are affected by sample size by calculating the statistical power for tau SUVR to distinguish FACs and NFACs. The statistical power ranged from 0.725 to 0.999 with power > 0.8 in 10 out of the 12 study ROIs. The statistical power for gray matter volume to distinguish between the two groups ranged from 0.220 to 0.999, with power > 0.8 in 5 out of the 12 study ROIs. This suggests that tau-PET may be more sensitive than spatial MRI to elucidate APOE ε4 and sex effects in AD.

Considering the exploratory nature of our study, our sample size and the correlated nature of the tau positive study ROIs 47, 48, we choose to minimize the probability of false negatives and include results without multiple comparisons correction in the main results. We recognize that this will increase our false positive rate and have evaluated robustness to false positives by applying the Benjamini-Hochberg method for ROI based analyses and family wise error (FWE) correction for voxelwise analyses. For the ROI-based ^18^F-AV-1451 analysis, all regions remained significant at false discovery rates (FDR) < 0.1 24. For the ROI-based gray matter analysis, three regions i.e. the amygdala, inferior temporal and posterior cingulate remained significant at FDR < 0.1. All regions remained significant at FDR < 0.2. For the voxelwise ^18^F-AV-1451 analysis, clusters in the right inferior temporal, left amygdala, inferior parietal, superior parietal, bilateral entorhinal cortex and parahippocampal gyrus regions remained significant after FWE correction. For the voxelwise gray matter analyses, no clusters survived FWE correction.

Further, we acknowledge that the current work is based on the ADNI cohort and may not generalize to other AD patient populations. Future analyses should be conducted in multi institutional cohorts beyond ADNI with larger sample sizes to validate this preliminary work.

## Conclusion

In summary, sex modifies the effects of the APOE ε4 allele on brain region-specific tau deposition and gray matter volume in older adults with AD. Specifically, FACs exhibited elevated brain region-specific tau PET SUVR and deceased gray matter volume. Results from this study advance our understanding of the effect of sex and APOE ε4 in promoting tau pathology in AD. Importantly, this work highlights the importance of considering sex and APOE ε4 in biomarker development, clinical trial endpoint evaluation, and mechanistic studies in AD using quantitative ^18^F-AV-1451 PET.

## Figures and Tables

**Table 1:** Study cohort characteristics

Parameter	Female APOE ε4+ (n = 15)	Female APOE ε4- (n = 10)	Male APOE ε4+ (n = 19)	Male APOE ε4- (n = 13)
Age (years)	76.58±7.98	79.26±11.60	79.32±8.63	81.06±9.86
Education (years)	14.73±1.94	13.50±2.01	17.11±2.62	16.00±2.71
MMSE	21.20±4.59	20.10±5.86	22.79±4.40	22.15±5.16
Global CDR	1.13±0.48	1.20±0.59	0.87±0.50	0.88±0.42
CDR-SOB	1.38±1.90	3.56±4.27	1.46±2.79	1.00±1.84
APOE ε4ε4/ε4ε3/ε4ε2	5/8/2	-	6/11/2	-
CSF Aβ42 (pg/mL)	589.56±180.47	795.35±262.64	625.24±195.16	722.17±343.62
CSF t-tau (pg/mL)	433.03±202.33	343.43±191.15	341.76±109.82	333.44±180.90
CSF p-tau (pg/mL)	43.31±25.37	31.60±21.84	34.19±11.86	33.34±21.58

Notes. CDR: Clinical Dementia Rating; CDR-SOB: Clinical Dementia Rating Scale sum of boxes; CSF: cerebrospinal fluid; MMSE: Mini-Mental State Examination.

**Table 2:** Clusters with increased tau deposition among FACs with AD

Clusters		Cluster size	Atlas Coordinates			Z score	*P* value
			X	Y	Z		
Middle temporal/ Middle temporal pole/Parahippocampal/Entorhinal cortex/Amygdala/Inferior parietal/Superior parietal/Superior temporal/Middle occipital/Precentral.	L	9892	-42	-54	60	6.27	< 0.001
Parahippocampal/Middle temporal pole/Inferior temporal/Middle temporal/Entorhinal cortex/Amygdala.	R	1020	16.5	-4.5	-21	5.87	< 0.001
Middle frontal/ Superior frontal/Middle orbitofrontal.	L	1416	-43	46	21	5.00	< 0.001

Notes: The data were extracted from voxels associated with maximally significant ^18^F-AV451 PET SUVR increases in FACs compared with the NFACs with AD, after controlling for age and years of education. Cluster locations correspond to the brain maps shown in Figure 3 and correspond to *P* < 0.001. Atlas coordinates were obtained from the AAL atlas. The X, Y and Z coordinates are shown in the MNI space. FACs: female APOE ε4 carriers.

**Table 3:** Clusters with decreased gray matter volume among FACs with AD

Clusters		Cluster size	Atlas coordinates (peak)			Z score	*P* value
			X	Y	Z		
Inferior temporal/ Occipital.	L	653	-54	-52.5	-22.5	-4.04	< 0.001
Superior temporal pole	R	135	58.5	15	-13.5	-3.78	< 0.001
Superior temporal	L	204	-63	-3	-9	-3.94	< 0.001
Middle temporal	L	446	-69	-30	1.5	-4.10	< 0.001
Amygdala	L	104	-17.5	-4.5	-16.5	-4.35	< 0.001
Middle occipital	L	137	-45	-82.5	10.5	-4.16	< 0.001
Superior occipital/ Precuneus.	R	762	27	-84	39	-4.73	< 0.001
Posterior cingulate	L	471	-3	-46.5	19	-5.71	< 0.001
Superior-medial frontal/Supplementary motor area/Superior frontal.	L/R	3003	1.5	54	42	-5.41	< 0.001

Notes: The data were extracted from voxels associated with maximally significant gray matter volume decreases in FACs compared to the NFACs with AD, after controlling for age and years of education. Listed locations correspond to the brain maps shown in Figure 5 and correspond to *P* < 0.001. Atlas coordinates were obtained from the AAL atlas. The X, Y and Z coordinates are shown in the MNI space. FACs: female APOE ε4 carriers, NFACs: non-FACs.

## Abbreviations

AD: Alzheimer's disease
ADNI: Alzheimer's disease neuroimaging initiative
APOE ε4: apolipoprotein E type 4 allele
CDR: Clinical Dementia Rating
CDR-SOB: Clinical Dementia Rating Scale sum of boxes
CSF: cerebrospinal fluid
FWHM: full width at half maximum
GLM: generalized linear model
MCI: mild cognitive impairment
MMSE: Mini-Mental State Examination
MNI: Montreal Neurological Institute
MRI: magnetic resonance imaging
PET: positron emission tomography
p-tau: phosphorylated tau
PVC: partial volume correction
ROI: region of interest
SAS: Statistical Analysis System
SPM: Statistical Parametric Mapping
SUVR: standardized uptake value ratio
t-tau: total tau
